# Histoplasmosis-Associated Hemophagocytic Lymphohistiocytosis: A Review of the Literature

**DOI:** 10.1155/2019/7107326

**Published:** 2019-10-01

**Authors:** Ra'ed Jabr, Wissam El Atrouni, Heather J. Male, Kassem A. Hammoud

**Affiliations:** ^1^Department of Internal Medicine, The University of Kansas Medical Center, Kansas City, KS 66160, USA; ^2^Division of Infectious Diseases, The University of Kansas Medical Center, Kansas City, KS 66160, USA; ^3^Division of Hematological Malignancies and Cellular Therapeutics, The University of Kansas Medical Center, Kansas City, KS 66160, USA

## Abstract

**Background:**

Histoplasmosis is an endemic fungal disease with diverse clinical presentations. Histoplasmosis-associated hemophagocytic lymphohistiocytosis (HLH) is a rare disorder with limited data regarding treatment and outcome. We described the clinical features, treatment, and outcomes of five patients in our institution with histoplasmosis-associated HLH. This review also summarizes the current literature about presentation, treatment, and outcome of this infection-related HLH entity.

**Methods:**

We searched the electronic medical records for patients with histoplasmosis-associated HLH at our institution from 1/1/2006 to 9/30/2017. Diagnosis of HLH was confirmed by chart review using the HLH-04 criteria. We also searched the current literature for case reports and case series.

**Results:**

Five cases of histoplasmosis-associated HLH were included from our institution. All five patients were diagnosed after 2010. The literature review yielded 60 additional cases of histoplasmosis-associated HLH. The most common underlying condition was HIV in 61% of cases. The majority of histoplasmosis patients (81%) were treated with amphotericin B formulations. Documented specific treatments for HLH were as follows: nine patients received steroids only, six patients received intravenous immunoglobulin (IVIG) only, three patients received dexamethasone and etoposide, two patients received etoposide, dexamethasone, and cyclosporine, two patients received steroids and IVIG, and one patient received Anakinra and IVIG. The inpatient case fatality rate was 31% with most of the deaths occurring within two weeks of hospital admission.

**Conclusions:**

Histoplasmosis-associated HLH among adults is an uncommon but serious complication with high associated mortality. Early antifungal therapy with a lipid formulation amphotericin B is critical. The initiation of immunosuppressive therapy with regimens like HLH-04 in this disease entity should be individualized.

## 1. Introduction

Hemophagocytic lymphohistiocytosis (HLH) is a rare but often life-threatening syndrome of excessive immune activation. Primary HLH is triggered by genetic disorders and usually manifests in children below the age of 18 months. The term secondary (acquired) HLH has generally been used to describe cases in adults without known genetic predisposition and a clear trigger for HLH. This trigger is often an infection or an alteration in immune homeostasis (e.g., malignancy, rheumatologic conditions, or immunodeficiency syndromes) [1]. HLH may present as a single episode of disease or recurrent episodes. Histoplasmosis-associated HLH is a relatively rare disorder with limited data about treatment [2, 3]. We describe the clinical features, treatment, and outcomes of cases seen at the University of Kansas Medical Center (KUMC). In addition, we reviewed the current literature about diagnosis and treatment of histoplasmosis-associated HLH.

## 2. Methods

We searched our medical informatics HERON (Healthcare Enterprise Repository for Ontological Narration) database for the following ICD9/ICD10 terms: “hemophagocytic syndrome,” “hemophagocytosis,” “hemophagocytic lymphohistiocytosis” or “macrophage activation syndrome” and “histoplasmosis” or “disseminated histoplasmosis.” We included patients older than 18 years and seen at our institution from 1/1/2006 to 9/30/2017. All patients who satisfied the HLH-04 criteria [4] for a diagnosis of HLH (i.e., 5 of 8 criteria) and confirmed to have histoplasmosis by retrospective chart review were included. HLH-04 criteria include (1) fever; (2) splenomegaly; (3) cytopenia in two or more cell lines; (4) hypertriglyceridemia (triglyceride level ≥265 mg/dL or hypofibrinogenemia (fibrinogen level ≤150 mg/dL); [5] hemophagocytosis in the bone marrow, spleen, or lymph nodes; [6] hyperferritinemia (ferritin level ≥500 ng/mL); [7] impaired NK cell function; and [8] elevated soluble CD25 (soluble IL-2 receptor alpha) two standard deviations above age-adjusted laboratory-specific norms. This study was approved by the University of Kansas Medical Center institutional board review.

## 3. Literature Review

We searched PubMed for “histoplasmosis” and “hemophagocytic syndrome,” “histoplasmosis” and “secondary hemophagocytic syndrome,” “histoplasmosis induced hemophagocytic syndrome,” “disseminated histoplasmosis” and “hemophagocytic syndrome,” “HLH” and “histoplasmosis,” and “histoplasma associated HLH.” Few papers were only available by a Google search. We included all case reports/series with a published abstract in English.

## 4. Results

We identified five cases of histoplasmosis-associated HLH. The clinical characteristics are summarized in Table 1. All patients had disseminated histoplasmosis and were immunosuppressed. Human immunodeficiency virus (HIV) infection was the underlying condition in 2/5 patients (40%); the diagnosis of HIV in both patients was new. The other three patients were on steroids in addition to other immunosuppressive agents. There was a male predominance 4/5 (80%). All patients 5/5 (100%) had positive blood cultures for histoplasma, and the histoplasma antigen (blood or urine) was above the limit of quantification. Histoplasma was seen on bone marrow (BM) biopsy in 3/4 (75%) patients.

Four patients (80%) met at least 5 out of 8 criteria for the diagnosis of HLH as shown in Table 2. The IL2-receptor, cytopenia, and ferritin criteria were met in all five patients. Peak ferritin level was above the limit of quantification in 4/5 (80%) patients. One patient met only four criteria, but the hematology consulting team felt that it was highly likely secondary HLH.

The treatment and outcomes are shown in Table 3. Most patients were started on liposomal amphotericin B for at least 2 weeks and then transitioned to an oral azole. One patient received only voriconazole. Three out of five patients survived to hospital discharge.

The literature review yielded 60 cases from 39 papers; most of them were case reports and few were case series.  Table 4 summarizes the patients baseline characteristics, treatment used, diagnostic tests for histoplasmosis, and outcomes. Five papers were published before 2000, and 18 papers were published since 2015. We report five patients at our institution from 1/1/2006 to 9/30/2017 (Table 1). All 5 cases at KUMC were diagnosed after 2010. Adding our five cases to the 60 reported previously, the median age of cases was 41 years and 72% (37/52) were men. The most common underlying immunosuppressive condition was HIV in 62% (36/58). Six patients had solid organ transplant, and there was no clear underlying immunodeficiency described in seven patients. In eleven patients, there was no mention of the host immune status.

The median CD4 count in HIV patients was 17 cells/*μ*L. The majority of patients had disseminated histoplasmosis. Five patients were diagnosed by either lymph node biopsy or histoplasma urine Ag only and not proven to have disseminated histoplasmosis.

Initial antifungal treatment consisted of amphotericin B formulation in 48 cases and only azoles in four cases. The specific treatment for HLH was as follows: nine patients received steroids only, six patients received intravenous immunoglobulin (IVIG) only, three patients received dexamethasone and etoposide, two patients received etoposide, dexamethasone, and cyclosporine, two patients received steroids and IVIG, and one patient received Anakinra and IVIG.

The overall inpatient case fatality rate was 31% (20/64) and 37% (13/35) among HIV patients. The mortality rate was 25.0% (12/48) in patients who received amphotericin B, 20% (1/5) in patients who received steroids and etoposide with or without cyclosporine (all received amphotericin B), 62% (5/8) in patients who received IVIG, and 31% (5/16) in patients who received steroids. One patient received Anakinra and IVIG and survived. Only 14/21 patients had available date of death; of those, 10 patients died within two weeks of admission and four patients died at hospital days 16, 18, 43, and 44.

## 5. Discussion

Histoplasmosis-associated HLH is rare but likely underdiagnosed given the nonspecific clinical and laboratory presentation. The diagnosis is challenging because high fever, peripheral blood cytopenia, splenomegaly, and elevated ferritin are very common in patients with disseminated histoplasmosis. We report the second largest case series of histoplasmosis-induced HLH. About half of the cases (29/60) were reported after 2014, and all five cases at our institution were diagnosed after 2010. This may be explained by an increased awareness of this entity. Almost all cases of histoplasmosis-associated HLH occurred in patients with disseminated histoplasmosis. Most patients were relatively young. HIV and its numerous related opportunistic infections remain the most common underlying immunodeficiency that triggers HLH, but the recent literature showed an increasing number of non-HIV patients (organ transplant, patients receiving chemotherapy, or other immunosuppressive treatments). This is likely caused by the recent increase in disseminated histoplasmosis among non-HIV infected patients [43, 44]. Only few patients had no clear underlying immunodeficiency. The male predominance may be related in part to the higher incidence and prevalence of HIV in men in the United States.

Human immunodeficiency virus (HIV) could trigger hemophagocytic syndrome by itself, or secondary to ART initiation or opportunistic infections [45]. In a retrospective study to evaluate the triggers of HLH among HIV patients in Brazil, opportunistic infections were the most common factors (59%) including *Mycobacterium* (34%), *Cytomegalovirus* (14%), and *Cryptococcus neoformans* (11%) [46].

Macrophage activation syndrome (MAS) is a form of HLH that is usually associated with rheumatologic diseases and inflammatory bowel diseases (IBD). In a review of literature of 50 cases of HLH or MAS, the association between HLH and IBD was thought to be secondary to infections, the effect of immunosuppressive therapy, and the potential presence of a genetic susceptibility [47]. The majority of cases were Crohn's disease (CD) rather than ulcerative colitis; this was attributed to the more frequent use of immunomodulators in CD.

The number of patients in each treatment group and the noncontrolled nature of this review hinder making conclusions about the most effective therapy. Patients who received amphotericin B had a slightly lower case fatality rate compared to the whole group. It is unclear if the addition of etoposide and steroids was helpful, but 4/5 patients with such regimen survived. We suggest starting a lipid formulation of amphotericin B as soon as possible, as recommended by the Infectious Diseases Society of America guidelines [48] for the treatment of moderate to severe disseminated histoplasmosis. There are limited data to establish the best treatment protocol and the role of immunosuppressive therapy and IVIG for histoplasma-associated HLH [1].

The treatment of secondary HLH is most effective when the inciting disease can be treated and controlled. If effective histoplasmosis treatment fails to demonstrate improvement after 48–72 hours, clinicians often consider initiation of immunosuppressive therapy with regimens such as the HLH-94 protocol [49]. Once clinical improvement is noted, we believe immunosuppressive therapy can be tapered, and the full protocol is often not required.

We report a small number of cases at our institution and it is possible that we could have missed cases in our retrospective search. In published case reports and case series, some data were lacking and few articles were not in English.

## 6. Conclusions

Histoplasmosis-associated HLH among adults is an uncommon but serious disease with high mortality. The clinical and laboratory findings that should prompt evaluation for HLH are splenomegaly, highly elevated ferritin, and cytopenia in an immunocompromised patient with disseminated histoplasmosis. The delay in diagnosis of HLH may affect outcomes and patients with suspected HLH should have a prompt hematology consultation. HLH appears to be a disease of excessive immune activation, and the optimal treatment and duration of immunosuppressive therapy remains unknown. Early antifungal therapy with a lipid formulation amphotericin B is critical. Multicenter prospective studies are needed to help define the role and duration of immunosuppressive therapy for this disease.

## Figures and Tables

**Table 1:** Clinical characteristics of histoplasmosis-induced HLH patients at our institution (*n* = 5).

Case #	Year	Age	Gender	Race	Comorbid conditions	Immunosuppressive agent	Yeast in BM	Urine histoplasma Ag	Sites growing histoplasma	CXR findings
1	2011	48	M	White	HIV/CD4 count 50	None	Yes	Above LoQ	Blood	No infiltrates
2	2014	48	F	White	MCTD	HCQ/prednisone 10 mg daily	No	Above LoQ	Blood	LAD without infiltrates
3	2016	75	M	White	Crohn's	Infliximab/azathioprine/prednisone	Yes	N/A (serum Ag: above LoQ)	Blood and BM	No infiltrates
4	2017	46	M	African American	Sarcoid	Prednisone	Yes	Above LoQ	Blood and BM	Diffuse infiltrate
5	2017	41	M	White	HIV/CD4 count 10	None	N/A	Above LoQ	Blood	Diffuse infiltrate

M: male; F: female; HIV: human immunodeficiency virus; MCTD: mixed connective tissue disease; N/A: not applicable, BM: bone marrow; dexa: dexamethasone; CXR: chest X-ray; Ag: antigen; LAD: lymphadenopathy; LoQ: limit of quantification; HCQ: hydroxychloroquine.

**Table 2:** Diagnosis of HLH (*n* = 5).

Case #	Fever	Cytopenia (2 lines)	IL2-receptor (pg/mL)	Peak triglycerides (mg/dL)	BM with hemophagocytosis	Splenomegaly	Peak ferritin (ng/mL)	Nadir fibrinogen (mg/dL)
1	Yes	Yes	5167	258	Yes	No	>15,000	95
2	Yes	Yes	115,900	329	Yes	Yes	4487	265
3	No	Yes	958	227	Yes	No	>7500	384
4	Yes	Yes	1648	192	No	Yes	>7500	168
5	No	Yes	15,540	246	N/A	Yes	>7500	51

**Table 3:** Treatment and outcome of histoplasmosis-associated HLH patients at our institution (*n* = 5).

Case #	Antifungal drug	HLH specific treatment	Outcome (hospital discharge)
1	Liposomal amphotericin B for 2 weeks, then itraconazole for 12 months	None	Survived
2	Liposomal amphotericin B for 4 weeks, then oral azoles for 4 years	Dexamethasone 10 mg/m^2^ for 2 days	Survived
3	Voriconazole PO (discharged to hospice, patient preference)	None	Discharged to hospice
4	Liposomal amphotericin B for 2 weeks then itraconazole for 4 months	Dexamethasone 10 mg/m^2^	Survived
5	Liposomal amphotericin B for 2 weeks then oral azoles	None	Died (day 43)

Notes. BM: bone marrow, IL: interleukin, N/A: not applicable.

**Table 4:** Cases of histoplasmosis-associated histiocytic lymphohistiocytosis (HLH) in the literature.

Author	Year	Country	Age, gender	Underlying disease	CD4	Treatment	Histoplasma diagnosis	Outcome^∗^
Majluf-Cruz et al. [5]	1993	Mexico	37 y, M	HIV	NR	Fluconazole	Liver Bx	Survived
			49 y, M	HIV	NR	Amphotericin B	BM Bx	Survived
			36 y, M	HIV	NR	None	BM Bx	Died (N/A)

Keller and Kurtzberg [6]	1994	USA	6 y	Chronic mucocutaneous candidiasis	N/A	Amphotericin B	BM Cx/blood Cx/BAL Cx	Survived

Koduri et al. [7]	1995	USA	NR	None	N/A	Amphotericin B/solumedrol	NR	Died (NR)

Koduri et al. [8]	1995	USA	NR	HIV	36	Amphotericin B/IVIG × 2d	Blood smear/BM path and Cx	Died (NR)
				HIV	4	Amphotericin B/IVIG × 2d	Blood smear/BM path/BM Cx/CSF Cx	Died (HD 6)
				HIV	6	Amphotericin B/IVIG × 2d	Blood smear/BM path and Cx	Died (NR)
				HIV	22	Amphotericin B/IVIG × 2d	BM path and Cx/skin Cx	Survived
				HIV	32	Amphotericin B	BM path and Cx	Survived
				HIV	44	Amphotericin B	BM path and Cx	Survived

Chemlal et al. [9]	1997	Africa	50 y	HIV	34	NR	Blood Cx/BM and skin path	NR

Kumar et al. [10]	2000	India	50 y, M	None	N/A	None	Splenic aspirate smears	Died (within 48 hours)

			40 y, M	HIV	NR	None	LN Bx	Died (within 48 hours)

Rao et al. [1]	2002	USA	68 y, M	CLL on cyclophosphamide and fludarabine	N/A	Amphotericin B	BM and lung Bx (path)	Survived

Masri et al. [11]	2003	USA	47 y, M	Heart transplant	N/A	Liposomal amphotericin B	BM (path and Cx)/peripheral smear/lung Bx (path and Cx)	Survived

Gil-Brusola et al. [12]	2007	Ecuador	33 y, M	HIV/disseminated TB	39	None	Blood smear/BM path	Died (HD 18)

Guiot et al. [13]	2007	Puerto Rico	43 y, M	HIV/ileal perforation	66	Liposomal amphotericin B for 21 days/itraconazole	GI (path)/BAL (cytology)/BM path and Cx, histoplasma PCR	Survived

Sanchez et al. [14]	2007	USA	61 y, M	HIV/pulmonary TB	4	Amphotericin B	Blood and BM (? Cx or path)	Survived

Wang et al. [15]	2007	USA	52 y, M	HCV, CKD	N/A	None (postmortem diagnosis)	Autopsy diagnosis/postmortem blood and spleen Cx	Died (HD 12)

Phillips et al. [16]	2008	USA	69 y, M	Sarcoidosis on chronic steroids	N/A	Amphotericin B/steroids/etoposide/cyclosporine	BM path and Cx/blood smear	Survived

De Lavaissiere et al. [17]	2009	France	33 y, M	HIV	NR	Amphotericin B/itraconazole/ART/IVIG	Blood and BM (Cx or path?)	Survived

Lo et al. [18]	2010	USA	22 y, F	Renal transplant	N/A	Liposomal amphotericin for 2 weeks/itraconazole	BM path/Blood Cx/urine Ag	Survived
		USA	18 y, M	Renal transplant	N/A	Liposomal amphotericin B for 1 week/itraconazole	LN path/BM Cx/urine Ag	Survived

van Koeveringe and Brouwer [19]	2010	Holland	50 y, M	CLL-alemtuzumab/fludarabine/cyclophosphamide	N/A	Dexamethasone/cyclosporine/etoposide/amphotericin B	BM path confirmed by PCR and culture	Survived

Vaid and Patel [20]	2011	UK	25 y, M	HIV	153	Antifungal	Skin/BM/oral mucosa path	Died (NR)

Chandra et al. [21]	2012	India	38 y, F	HIV	NR	Ketoconazole	NR	Survived

Nieto-Rios et al. [22]	2012	Colombia	30 y, F	Renal transplant	N/A	Amphotericin B/itraconazole	Blood Cx	Survived

			41 y, F	Renal transplant	N/A	None (died the same day of positive Cx)	Blood Cx	Died (HD 3)

Telfer and Gulati [23]	2012	USA	28 y, M	HIV	12	Voriconazole	BM path/urine Ag	Died (NR)

Huang [24]	2014	Guatemala	25 y, M	HIV	4	Antifungals/dexamethasone	Urine Ag/BM path	Survived

Subedee and van Sickels [25]	2015	USA	42 y, F	HIV	40	Liposomal amphotericin B/itraconazole	Urine Ag/BM path	Survived

Rajput et al. [26]	2015	Canada	64 y, F.	CKD, SCD	N/A	Antifungal therapy	BM and blood Cx	Survived

Kashifet al. [27]	2015	USA	34 y, M	SCD	N/A	Amphotericin B/itraconazole/dexamethasone/etoposide	LN Bx	Died (HD 8)

Castelliet al. [28]	2015	Mexico	32 y, M	HIV	3	Liposomal amphotericin B × 2 weeks/itraconazole/dexamethasone/etoposide	BAL Cx/Blood Cx/BM Cx	Survived

Mukherjee and Basu [29]	2015	India	52, M	COPD, Type II DM	N/A	Amphotericin B	Autopsy	Died (NR)

Townsend et al. [30]	2015	Mexico	31 y, F	HIV	1	Liposomal amphotericin B	Blood and BM Cx	Died (HD 16)
		USA	53 y, M	HIV	6	Liposomal amphotericin B × 14 d/itraconazole	Urine Ag	Survived
		USA	33 y, F	HIV	1	Liposomal amphotericin B × 21 d/fluconazole	Blood Cx/urine Ag	Survived
		Mexico	47 y, M	Immunosuppressive treatment		Itraconazole	Urine Ag	Survived
		USA	28 y, M	HIV	N/A	NR	Blood Cx/BM Cx/urine Ag	Survived
		USA	60 y, M	Immunosuppressive treatment	N/A	Liposomal amphotericin B/voriconazole/steroids/tacrolimus	BM path/skin Bx/urine Ag	Survived
		USA	44 y, M	HIV	2	Liposomal amphotericin B × 16 d/itraconazole	Sputum and blood Cx/urine Ag	Survived
		USA	52 y, M	HIV	16	Liposomal amphotericin B × 6 d/itraconazole/steroids/IVIG	BM Cx and urine Ag	Died (HD 9)
		USA	52 y, M	HIV	16	Liposomal amphotericin B × 3 d/itraconazole/IVIG	Blood Cx/BM path/urine Ag	Died (HD 9)
		El Salvador	32 y, M	HIV	50	Liposomal amphotericin B × 18 d/itraconazole	Blood, sputum, and BM Cx	Survived
		USA	51 y, M	HIV	9	Liposomal amphotericin B/steroids	Sputum, blood, gastric tissue Cx	Died (HD 13)

De and Nath [31]	2015	India	40 y, M	Healthy	N/A	Amphotericin B deoxy/itraconazole	BM Bx	Survived
			32 y, M	Healthy	N/A	Amphotericin B/itraconazole	BM Bx	Survived

Sonavane et al. [32]	2016	India	43 y, F	Healthy	N/A	Amphotericin B/itraconazole	BM Bx	Survived

Nieto et al. [33]	2016	Colombia	33 y, M	HIV	N/A	Amphotericin B/steroids	NR	Survived

Ferguson-Paulet al. [34]	2016	USA	6 months, F	MRSA bacteremia	N/A	Liposomal amphotericin B/itraconazole/etoposide/steroids	Urine serum Ag/BM and CSF Cx	Survived

Daoand He [35]	2016	USA	21 y, M	Crohn disease	N/A	Antifungal therapy	BM (cx and path)/Blood and BAL Cx	Survived

Schulze et al. [36]	2016	Germany	59 y, F	Steroids for suspected IBD	N/A	Liposomal amphotericin B/posaconazole/steroids	Colon, liver, LN, lung path/blood Cx	Survived

Gómez-Espejo et al. [37]	2017	Venezuela	23 y, M	HIV	7	Liposomal amphotericin B/dexamethasone/IVIG	Liver Bx	Survived

Karthik Bommanan et al. [38]	2017	India	32 y, M	Healthy	N/A	Amphotericin B	BM Bx path	Survived

Souto Filho et al. [39]	2017	Brazil	40 y, F	SLE	N/A	Amphotericin B/steroids	BM Bx path	Died (within 48 hrs)

Ocon et al. [40]	2017	Guyana	49 y, M	HIV	7	Liposomal amphotericin B/ART/IVIG/anakinra	Blood cx	Survived

Loganantharajet al. [41]	2017	Dominican Republic	46 y, M	HIV	54	Liposomal amphotericin B	Urine Ag/LN bx	Survived

Huapaya et al. [42]	2017	USA	46 y, M	Kidney transplant	N/A	Amphotericin B	Urine and serum Ag/BAL cytology	Survived

Abbreviations: y, year; M, male; F, female; HIV, human immunodeficiency virus; NR, not reported; BM, bone marrow; N/A, not applicable; BAL, bronchoalveolar lavage; ART, antiretroviral therapy; IVIG, intravenous immunoglobulin; d, days; wks, weeks; Cx, culture, Bx, biopsy; path, histopathology; CSF, cerebrospinal fluid; CLL, chronic lymphocytic leukemia, TB, tuberculosis; GI, gastrointestinal; PCR, polymerase chain reaction; HCV, hepatitis C virus; CKD, chronic kidney disease; Ag, antigen; LN, lymph node; MCTD, mixed connective tissue disease; HD, hospital day; IBD: inflammatory bowel disease.
